# The role of reactive oxygen species and proinflammatory cytokines in type 1 diabetes pathogenesis

**DOI:** 10.1111/j.1749-6632.2012.06826.x

**Published:** 2013-01-16

**Authors:** Lindsey E Padgett, Katarzyna A Broniowska, Polly A Hansen, John A Corbett, Hubert M Tse

**Affiliations:** 1 Department of Microbiology, Comprehensive Diabetes Center, University of Alabama at BirminghamBirmingham, Alabama; 2 Department of Biochemistry, The Medical College of WisconsinMilwaukee, Wisconsin

**Keywords:** cytokines, reactive oxygen species, type 1 diabetes

## Abstract

Type 1 diabetes (T1D) is a T cell–mediated autoimmune disease characterized by the destruction of insulin-secreting pancreatic β cells. In humans with T1D and in nonobese diabetic (NOD) mice (a murine model for human T1D), autoreactive T cells cause β-cell destruction, as transfer or deletion of these cells induces or prevents disease, respectively. CD4^+^ and CD8^+^ T cells use distinct effector mechanisms and act at different stages throughout T1D to fuel pancreatic β-cell destruction and disease pathogenesis. While these adaptive immune cells employ distinct mechanisms for β-cell destruction, one central means for enhancing their autoreactivity is by the secretion of proinflammatory cytokines, such as IFN-γ, TNF-α, and IL-1. In addition to their production by diabetogenic T cells, proinflammatory cytokines are induced by reactive oxygen species (ROS) via redox-dependent signaling pathways. Highly reactive molecules, proinflammatory cytokines are produced upon lymphocyte infiltration into pancreatic islets and induce disease pathogenicity by directly killing β cells, which characteristically possess low levels of antioxidant defense enzymes. In addition to β-cell destruction, proinflammatory cytokines are necessary for efficient adaptive immune maturation, and in the context of T1D they exacerbate autoimmunity by intensifying adaptive immune responses. The first half of this review discusses the mechanisms by which autoreactive T cells induce T1D pathogenesis and the importance of ROS for efficient adaptive immune activation, which, in the context of T1D, exacerbates autoimmunity. The second half provides a comprehensive and detailed analysis of (1) the mechanisms by which cytokines such as IL-1 and IFN-γ influence islet insulin secretion and apoptosis and (2) the key free radicals and transcription factors that control these processes.

## Type 1 diabetes: a T cell–mediated autoimmune disease

Type 1 diabetes **(**T1D) is a polygenic autoimmune disease characterized by the destruction of insulin-secreting pancreatic β cells. Precipitating from a loss of self-tolerance, adaptive immune lymphocytes, such as CD4^+^ and CD8^+^ T cells, in addition to B cells, play a prominent role in β-cell destruction.^1^–^5^ With disease incidence climbing steadily at a rate of 3% per year and a concordance rate of 40–60% for monozygotic twins, genetics cannot be the only contributing factor to disease onset.^6^–^8^ Until insulin was discovered as the hormone responsible for maintaining glucose homeostasis in the 1920s, T1D was a lethal disease; but despite the current widespread use of exogenous insulin to maintain normal blood glucose, insulin insufficiency in vital organs leads to numerous life-threatening complications, including nephrophathy, neuropathy, and retinopathy.^9^ While innate immune cells, such as macrophages, and soluble mediators, such as chemokines, are also acknowledged as essential contributors of disease onset, much interest has focused on the T cell for numerous reasons. First, the islets of humans T1D patients in early stages of disease onset reveal a sparse infiltrate dominated by CD8^+^ T cells.^10^ In addition, as disease progresses, an increase in lymphocyte infiltrate usually correlates with decreased β-cell mass, as insulin staining is negative in islets in which a massive lymphocyte infiltrate is present.^10^ Finally, the most successful immunological therapies for T1D have been those that target autoreactive T cells (e.g., cyclosporine A, monoclonal anti-CD3, thymoglobulin).^11^–^15^ The first half of this review focuses on the role of diabetogenic T cells in T1D pathogenesis and the mechanisms by which they destroy pancreatic β cells, including proinflammatory cytokine synthesis. In addition, we also discuss how ROS influence adaptive immunity by stimulating the production of proinflammatory cytokines and the experimental tools used to explore the role of ROS in adaptive immunity.

## The importance of CD8^+^ T cells in T1D pathogenesis

**Figure 1:**
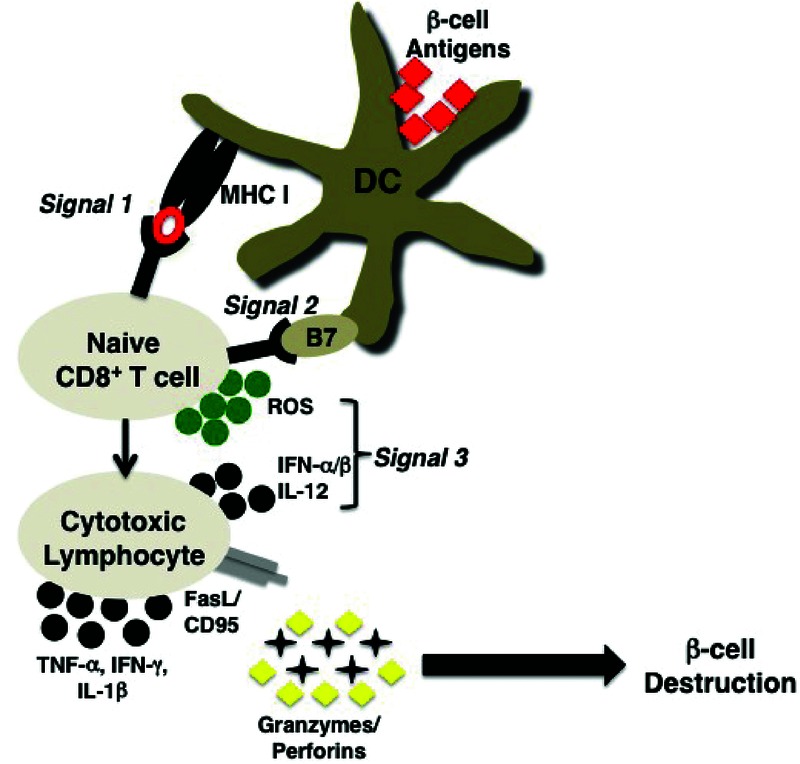
ROS-derived proinflammatory cytokines provide a third signal for optimal effector responses of naive CD8^+^ T cells. Cross-presentation of β-cell antigens to naive CD8^+^ T cells in the presence of ROS and proinflammatory cytokines IL-12 and IFN-α/β will facilitate the maturation of CTLs. Pancreatic β cells will be destroyed by CTLs by FasL (CD95), which upon binding to Fas expressed on the surface of β cells induces death, in addition to secretion of granzyme and perforin and the proinflammatory cytokines TNF-α, IFN-γ, and IL-1β.

Diabetogenic CD8^+^ T cells possess a prominent role in the pathogenesis of T1D, as NOD mice deficient in CD8^+^ T cells do not develop autoimmunity.^16^–^18^ Due to their cytotoxic properties, CD8^+^ T cells are widely acknowledged as the most important killer of human islet β cells in T1D.^19^,^20^ The elucidation of various murine CD8^+^ T cell autoreactive clones with distinct autoantigenic specificities, including G9C8,^21^ NY8.3,^22^,^23^ and AI4,^22^ which recognize insulin, islet-specific glucose-6-phosphatase catalytic subunit-related protein (IGRP) and dystrophia myotonica kinase (DMK), respectively, in addition to the CD8^+^ TCR transgenic strains employing these clones,^24^–^26^ have enabled researchers to thoroughly examine the role of CD8^+^ T cells in disease pathogenesis. Autoreactive CD8^+^ T cells induce β-cell destruction upon activation via MHC class I engagement on β cells during the effector stage of disease and are essential in priming and expanding diabetogenic CD4^+^ T cells.^27^ Effector CD8^+^ T cells, or cytotoxic T lymphocytes (CTLs), bring about β-cell death by releasing cytotoxic granules, such as perforins and granzymes, via exocytosis after direct contact with target cells (Fig. 1).^28^ Upon their release, perforins create holes in the plasma membrane of islet cells, allowing cytotoxic serine proteases (granzymes) to enter and induce cell death by apoptotic and necrotic pathways.^28^ In addition to the release of perforins and granzymes, CTLs also kill by upregulating Fas ligand (FasL) upon direct contact with β cells, thereby initiating apoptosis (Fig. 1).^29^

While CD8^+^ T cells play a vital role in T1D pathogenesis, these cytotoxic T cells do not account for all means of β-cell destruction, as perforin-deficient NOD mice are not fully protected against disease onset, and blocking Fas signaling within β cells provides only partial protection against T1D.^29^ NOD mice lacking MHC class I expression by β cells still develop early insulitis, but the incidence of T1D and hyperglycemia is significantly reduced, suggesting that MHC class I–dependent cytotoxicity of β cells is a late event in disease progression.^30^ In contrast, studies using an α-CD8–depleting antibody indicated that CD8^+^ T cells act in the preinsulitic stage, as depletion of these cells at two to five weeks within NOD mice protected against T1D onset; however, when depletion occurred at seven weeks of age, NOD mice succumbed to disease similar to controls.^27^ Hence, while the timing at which CD8^+^ T cells are most pathogenic in the context of pancreatic β-cell destruction remains unknown, ultimately, the remaining diabetes that occurs within these different systems is likely due to CD4^+^ T cells, which also induce T1D pathogenesis via β-cell destruction but by distinctly different means than CD8^+^ T cells.

## The importance of CD4^+^ T cells in T1D pathogenesis

CD4^+^ T cells play a critical role in T1D disease pathogenesis, as NOD mice deficient in CD4^+^ T cells are completely protected against spontaneous disease onset.^31^ Similar to the elucidation of the role of cytotoxic CD8^+^ T cells in T1D pathogenicity, the creation of a panel of highly diabetogenic CD4^+^ T cell clones from pancreatic lymph nodes of diabetic NOD mice with distinct autoantigenic specificities and, in turn, TCR transgenic models has allowed for detailed examination of the role of CD4^+^ T cells in disease pathogenesis.^32^–^34^ In particular, the diabetogenic CD4^+^ T cell clone BDC-2.5, which recognizes the β-cell secretory protein chromogranin A, and the NOD. BDC-2.5 mouse strain have been invaluable tools for dissecting the role of a specific CD4^+^ T cell clone in β-cell destruction and T1D pathogenesis.^33^,^34^

Interestingly, previous studies have established that CD4^+^ T cells alone can invade NOD pancreatic islets, while CD8^+^ T cells do not enter islets unless CD4^+^ T cells are also present.^35^–^37^ CD4^+^ T cells act both early and late in T1D pathogenesis, as adoptive transfer of CD4^+^ T cells precipitates disease, and depletion of CD4^+^ T cells protects against disease onset.^31^ Unlike CD8^+^ T cells, diabetogenic CD4^+^ T cells do not kill autoantigenic targets via direct contact, nor does killing involve Fas/FasL signaling and perforin, as adoptive transfer of NOD.BDC-2.5 CD4^+^ T cells into NOD mice lacking β cell–specific expression of Fas fails to protect against disease onset.^38^ Instead, autoreactive CD4^+^ T cells synthesize proinflammatory cytokines such as IFN-γ and TNF-α for β-cell destruction.

## The importance of proinflammatory cytokines in autoimmune diabetes pathogenesis

Synthesis of the proinflammatory cytokine IFN-γ by diabetogenic T cells has been classically acknowledged as an important contributor to autoimmune pathogenesis in T1D.^39^ Blocking IFN-γ within NOD mice via IFN-γ–specific antibodies^40^ or soluble IFN-γ receptors^41^ significantly reduces spontaneous T1D and prevents adoptive transfer of disease, while transgenic expression of IFN-γ within β cells exacerbates autoimmunity.^42^ Nevertheless, the mechanism by which IFN-γ induces autoimmunity in T1D has not been fully elucidated, as the onset of T1D is not significantly affected in NOD mice genetically lacking expression of IFN-γ^43^ and/or IFN-γR^44^,^45^ within β cells.

**Figure 2:**
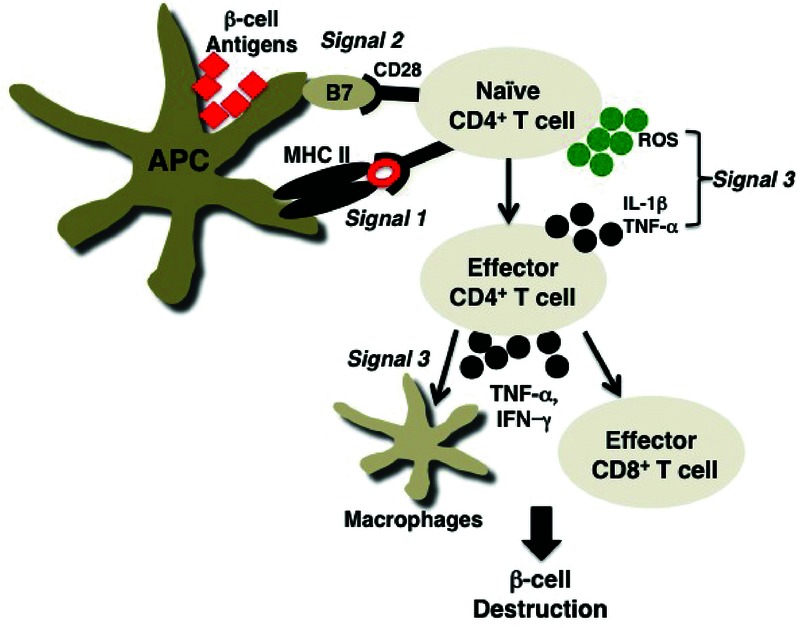
ROS-derived proinflammatory cytokines provide a third signal for optimal effector responses of naive CD4^+^ T cells. Naive CD4^+^ T cells mature into potent effectors of β cell destruction when stimulated with processed pancreatic β-cell epitopes presented on MHC II molecules, costimulatory molecules, and soluble mediators such as ROS, IL-1, and TNF-α. Activated effector CD4^+^ T cells will secrete the pleiotropic proinflammatory cytokine TNF-α, in addition to IFN-γ, to directly destroy β cells and/or facilitate the recruitment and activation of accessory cells, such as macrophages, NK cells, or CTLs, to mediate pancreatic β-cell destruction.

An important effector of T1D pathogenesis, TNF-α promotes cell adhesion by endothelial cell activation,^46^,^47^ leukocyte homing,^48^ upregulation of MHC class I and II within the islet,^49^,^50^ and activation of T cells and antigen-presenting cells (APCs). Production of TNF-α by CD8^+^ T cells is directly cytotoxic to β cells.^38^,^51^ CD8^+^ T cells secrete IFN-γ and TNF-α to upregulate autoantigen presentation on dendritic cells (DCs) and to enhance Fas and MHC class I expression on β cells, thereby augmenting T cell–mediated autoimmunity and promoting β-cell destruction.^52^ The CD8^+^ T cell clone G9C8 requires TNF-α for β-cell destruction, as adoptive transfer of G9C8 CD8^+^ T cells into NOD.TNFR1/2 KO recipients induced significant delays in T1D onset compared to NOD.^38^ Like CD8^+^ T cells, CD4^+^ T cells synthesize the proinflammatory cytokine TNF-α for efficient and effective β-cell destruction, as adoptive transfer of diabetogenic NOD.BDC-2.5 CD4^+^ T cells into NOD mice deficient in TNF receptor 1 and 2 (TNFR1/2) significantly delays T1D onset.^38^ Unlike CD8^+^ T cells, CD4^+^ T cell production of TNF-α activates dendritic cells, natural killer cells, and macrophages to propagate β-cell destruction (Fig. 2). In particular, BDC-2.5 CD4^+^ T cells initiate β-cell destruction by recruiting proinflammatory macrophages into the pancreas, as shown by adoptive transfer of the transgenic CD4^+^ T cells into immune deficient hosts.^53^ Thus, further studies are warranted to elucidate the individual roles of CD8^+^ T cells, macrophages, dendritic cells, and natural killer cells in TNF-α–dependent pancreatic β-cell destruction by CD4^+^ T cells.

## The many roles of ROS

The scope of ROS involvement in key biological functions has widened dramatically. Classically regarded to possess direct, potent antimicrobial properties, ROS influence a variety of essential processes, including signaling, autophagy, chemotaxis, antigen cross-presentation, and immune modulation.^54^ The importance of ROS in eradicating pathogens is evident in chronic granulomatous disease (CGD) patients who possess increased susceptibility to severe, recurrent bacterial and fungal infections due to defective NOX activity.^55^,^56^ In addition to antimicrobial responses, high localized concentrations of ROS (superoxide, hydrogen peroxide, hydroxyl radicals) and/or decreased expression of antioxidants (superoxide dismutase, glutathione peroxidase, catalase) can induce oxidative stress states, which, in turn, propagate pathological tissue destruction of various autoimmune and inflammatory diseases, such as rheumatoid arthritis (RA),^57^,^58^ multiple sclerosis,^59^,^60^ inflammatory bowel disease (IBD),^61^ atherosclerosis,^62^ and T1D.^2^ Illustrative of the role of oxidative stress in T1D, a recent study concluded that sera from T1D patients exhibited enhanced reactivity with hydroxyl radical–modified glutamic acid decarboxylase 65 (GAD65), a putative autoantigen in T1D pathogenesis detected in 60% of newly diagnosed patients.^63^,^64^ Interestingly, recognition of hydroxyl radical–modified epitopes of GAD65 was even more pronounced with sera from patients suffering from the diabetic non-diabetes complications retinopathy and nephropathy, indicating that oxidation by ROS may generate highly immunogenic neoepitopes that drive T1D pathogenesis.^65^ Unlike conditions of chronic oxidative stress, acute fluctuations in ROS and/or transient synthesis of these highly reactive molecules function as inter- and intracellular signaling second messengers.^66^,^67^

## ROS and the third signal for adaptive immune maturation

In addition to inducing oxidative stress and acting as second messengers, ROS potently affect adaptive immune maturation by promoting the synthesis of proinflammatory cytokines, such as TNF-α and IL-1β, by macrophages and dendritic cells via the mitogen-activated protein kinase (MAPK), activator protein-1 (AP-1), and nuclear factor kappa light chain enhancer of activated B cells (NF-κB) pathways.^3^–^5^,^68^–^74^ Proinflammatory cytokines induce efficient adaptive immunity, and in the case of T1D they facilitate the demise of insulin-secreting pancreatic β cells.^4^,^75^ Thus, ROS and innate-derived proinflammatory cytokines synergize to generate a productive immune response by linking innate with adaptive immune mechanisms.^5^,^74^

To demonstrate the influence of ROS on T cell responses studies by Jackson *et al.* revealed the rapid upregulation of hydrogen peroxide and other members of the NOX-derived ROS family by stimulated CD4^+^ T cells.^76^ ROS and proinflammatory cytokines collectively act as a third signal for efficient immune activation, in which the first signal involves antigen presented to the T cell receptor (TCR) in the context of MHC class I or II and the second signal comprises costimulatory molecule interactions.^77^,^78^ Studies have concluded that signals 1 and 2 are not sufficient for full activation of effector CD8^+^ and CD4^+^ T cell subsets;^3^,^4^ and while antigen presentation and costimulation promote naive T cell proliferation, these signals are collectively ineffectual at inducing adequate survival, optimal effector responses, and formation of memory T cell populations.^79^ Thus, ROS-derived proinflammatory cytokines provide the third signal for inducing a productive immune response by promoting survival, potent effector function, and T cell memory.^80^

## Proinflammatory cytokines and the third signal for CD4^+^ and CD8^+^ T cells

As T cells propagate T1D pathogenesis, insight into the mechanism by which they mature and become effectors of β-cell destruction is vital. As previously stated, ROS and, in turn, proinflammatory cytokines collectively provide a third signal for efficient adaptive immune maturation. While ROS generate efficient adaptive immunity by participating in redox-dependent signaling cascades, proinflammatory cytokines act differently to promote efficient adaptive immunity. Notably, IL-12 and type I interferons (IFNs; IFN-α/β) are necessary for maturing CD8^+^ T cell cytotoxic lymphocyte responses (Fig. 1),^4^,^81^ and IL-1β has a profound role in the effector response of CD4^+^ T cells (Fig. 2).^3^,^82^

## IL-12 and IFN-α/β act as third signals for CD8^+^ T cell–adaptive immune maturation

Seminal studies by Curtsinger *et al.* utilizing artificial APCs offered initial evidence that IL-12 and IFN-α/β were the key third signal proinflammatory cytokines for CD8^+^ T cells by upregulating IFN-γ production, promoting memory, inducing cytolytic activity, and increasing the rate of clonal expansion.^3^,^81^ Moreover, *in vivo* studies revealed that a cocktail of IL-12 and IFN-α/β replaced the need for adjuvant in peptide immunization models. Gene expression studies performed to elucidate the molecular mechanism of ROS-derived signal 3 proinflammatory cytokines revealed that gene expression levels altered by IL-12 and IFN-α/β included genes with products involved in cytolytic effector functions (granzymes, FasL, IFN-γ), proliferation, costimulation (CD25, OX40, 4–1BB), survival (serine protease inhibitor 6, Bcl-3), and trafficking/migration.^83^–^85^ With only signals 1 and 2, gene expression was rapidly upregulated but quickly declined to almost baseline levels; but in the presence of IL-12 and IFN-α/β, gene expression was elevated and sustained. As transcript levels were quenched in the absence of IL-12 and IFN-α/β, it was hypothesized that these proinflammatory cytokines induced chromatin remodeling. Further studies identified this as the mechanistic basis of signal 3 CD8^+^ T cell differentiation; for example, signals 1 and 2 combined with histone deacetylase inhibitors mimick the effects of IL-12 and type I IFNs on CD8^+^ T cell effector responses.^83^

## IL-1 acts as a third signal for efficient CD4^+^ T cell–adaptive immune maturation

While the ROS-derived proinflammatory cytokines IL-12 and IFN-α/β provide the third signal for CD8^+^ T cell clonal expansion, memory, and efficient cytolytic activity, IL-1 has been shown to provide the proinflammatory third signal for CD4^+^ T cells.^3^,^4^,^86^ In particular, proliferation of CD4^+^ T cells increases twofold with the addition of IL-1-α/β in the presence of antigen (signal 1) and costimulation (signal 2).^3^

In the context of T1D, experiments with IL-1 receptor (IL-1R)–deficient NOD mice have demonstrated the importance of IL-1 in autoimmune diabetes.^87^ A significant delay in T1D was observed with NOD mice lacking IL-1R expression, in contrast to that observed in wild-type NOD mice. In addition, the importance of this cytokine as a proinflammatory third signal for maturing autoreactive CD4^+^ and CD8^+^ T cell responses was evident, as diabetogenic NOD.NY8.3 CD8^+^ TCR transgenic mice lacking IL-1R expression were still capable of transferring T1D.^87^ However, when diabetogenic NOD.BDC-2.5 CD4^+^ T cells were transferred to IL-1R–deficient NOD mice, a delay in the onset of T1D was observed, further suggesting the importance of IL-1 to efficiently mediate autoreactive CD4^+^, but not CD8^+^, adaptive immune responses. While IL-1 has been shown to act directly as a third signal proinflammatory cytokine for CD4^+^ T cells, the molecular mechanism of CD4^+^ T cell immune maturation remains largely unknown.^82^ However, in experimental autoimmune encephalomyelitis (EAE), a mouse model for multiple sclerosis,^88^,^89^ the synthesis of IL-1 is necessary for autoreactive T cell induction and EAE development.^90^ The resistance to EAE in IL-1–deficient mice was partly attributed to inefficient generation of Th1 and Th17 T cell responses; and previous results have shown that IL-1 synthesis by APCs is necessary to facilitate efficient T cell interactions by enhancing T cell expression of CD154 (CD40L) and CD134 (OX40) to induce TNF-α synthesis and adaptive immune maturation.^91^,^92^ Thus, similar to the third signal cytokines IL-12 and type I IFNs, IL-1 may function to induce antigen-specific activation of CD4^+^ T cells by influencing chromatin remodeling. Future studies should be performed to elucidate the molecular mechanism by which IL-1 influences CD4^+^ T cell third signal activity.

## ROS modulation of T cell responses using SOD mimetics

As ROS are essential for initiating proinflammatory cytokine production and adaptive immune maturation, the generation of these highly reactive molecules should be regarded as a necessary proinflammatory-derived third signal for mediating effective adaptive immune activation. Redox modulation profoundly affects T cell responses, which has been illustrated with metalloporphyrin-based superoxide dismutase (SOD) mimetics that scavenge a wide array of ROS. Analysis of CD8^+^ T cell responses following treatment of lymphocyte choriomeningitis virus (LCMV)-infected mice with a SOD mimetic revealed significant reductions in circulating antigen–specific CD8^+^ T cells compared with vehicle treatment, indicating that ROS are critical for CD8^+^ T cell proliferation and antigen-specific clonal expansion.^154^ Further validating the crucial role of ROS in CD8^+^ T cell activity, Sklavos *et al.* demonstrated that ablation of free radicals via a SOD mimetic inhibited CD8^+^ T cell proliferation, proinflammatory cytokine production, and CTL target lysis, concomitant with profound reductions in perforin and granzyme B.^73^ In addition to affecting CD8^+^ T cells, a SOD mimetic induced CD4^+^ T cell antigen–specific hyporesponsiveness and reduced effector responses within polyclonal (BALB/c), autoimmune-prone (NOD), TCR transgenic (DO11.10, OT-II), and diabetogenic TCR transgenic (NOD.BDC-2.5) mouse strains.^74^ Importantly, SOD mimetic treatment of immunodeficient mice adoptively transferred with the BDC-2.5 CD4^+^ T cell clone provided significant protection against T1D by blunting IFN-γ production and T cell proliferation, illustrating the ability of ROS to modulate adaptive immune responses by influencing proinflammatory cytokine production.^93^ Thus, ablation of ROS reveals the potent effects of these highly reactive molecules on efficient adaptive immune maturation.

## The NOD.*Ncf1^m1J^* mouse strain

To further explore the role of ROS in modulating autoreactive T cell effector responses, the NOD.*Ncf1^m1J^*mouse strain was generated;^85^ this strain expresses a dominant negative mutation in the p47*^phox^* subunit (*Ncf1^m1J^*) that is essential for NOX assembly. Normally localized in the cytosol in a quiescent state, p47*^phox^* is hyperphosphorylated upon NOX activation and migrates to the membrane, where it associates with other components of the NOX multisubunit enzyme to generate superoxide.^195^ Interestingly, possessing a deficiency in NOX-derived superoxide, NOD.*Ncf1^m1J^* mice were highly protected from spontaneous diabetes.^85^ Analysis of *in vitro* NOD.*Ncf1^m1J^* T cell responses indicated that ablation of NOX-derived superoxide profoundly affected proinflammatory cytokine production and adaptive immune effector responses.^85^,^94^ Specifically, dampened Th1 (IFN-γ) and elevated Th17 (IL-17A) CD4^+^ T cell responses were observed upon polyclonal stimulation of NOD.*Ncf1^m1J^*CD4^+^ T cells, in addition to reduced IFN-γ production by NOD.*Ncf1^m1J^* CD8^+^ T cells.^85^,^94^ Granzyme production by NOD.*Ncf1^m1J^* CD8^+^ T cells was also significantly reduced compared to NOD CD8^+^ T cells, indicating that NOX synthesis was necessary for efficient CTL effector responses.^94^ In addition, adoptive transfer of NOD.*Ncf1^m1J^* T cells into NOD.*scid* recipients elicited a significant delay in T1D (compared with NOD T cells), indicating that NOX-derived superoxide was essential for induction of robust adaptive immunity.^94^ Interestingly, adoptive transfers of NOX-deficient CD4^+^ T cells with NOX-sufficient CD8^+^ T cells induced disease more rapidly than transfer of NOX-deficient CD8^+^ T cells with NOX-sufficient CD4^+^ T cells, indicating the importance of NOX in maturing autoreactive CTL effector responses during pancreatic β-cell destruction.^156^

## Other strains employing the *Ncf1^m1J^* mutation

As additional information is acquired about ROS and their role in adaptive immune activation, we are beginning to realize that immune modulation by ROS is complex, due in part to their dependence on localized concentration and environment. Illustrative of this complex role of ROS in immune activation, the *Ncf1^m1J^* mutation was shown to induce enhanced disease severity in rodent models of RA and EAE.^88^,^89^ In addition to the enhanced proclivity for autoimmunity, these superoxide-deficient rodents produced elevated levels of the proinflammatory cytokines IFN-γ,^88^ TNF-α,^88^ and IL-17.^95^ Moreover, despite exhibiting reduced synthesis of Th1 CD4^+^ T cell responses and reduced CD8^+^ CTL activity, the NOD.*Ncf1^m1J^* mouse strain displayed enhanced sensitivity to myelin oligodendrocyte glycoprotein (MOG)–induced EAE, concomitant with enhanced CD4^+^ T cell IL-17 production upon polyclonal stimulation.^85^ In addition to functioning as an innate immune–derived third signal for efficient maturation of β cell–specific autoreactive T cell responses in T1D, ROS synthesis may also be involved in peripheral tolerance and regulatory T (T_reg_) cell function in other autoimmune diseases.^96^ Indeed, analyses of various rodent models of arthritis, which collectively display enhanced susceptibility to autoimmunity with the *Ncf1^m1J^* mutation, reveal dampened T_reg_ cell induction and exacerbated autoimmune-mediated destruction of joints and tissues in the absence of superoxide.^97^ Specifically, induction of peripheral T_reg_ cells required NOX-sufficient macrophages; similarly, macrophages from CGD patients exhibit defects in T_reg_ cell induction and T cell suppressive function in contrast to those derived from healthy controls.^97^ Moreover, studies on the influence of the *Ncf1^m1J^* mutation on T_reg_ cell–mediated suppression of T cell responses have indicated that ablation of NOX-derived superoxide renders T_reg_ cells less effective at suppressing immune responses.^96^ This dichotomous role of ROS in protecting against autoimmunity in certain autoimmune-prone settings and enhancing adaptive immune maturation in other settings could be due to a variety of factors. In particular, the inherent levels of ROS produced and the sensitivity of immune cells to redox-dependent signaling within these various strains could be different, thereby differentially influencing the effector responses of T cells and mediating tolerance. Moreover, organ-specific or systemic autoimmunity within these mouse strains, in addition to different genetic make-up, may influence the actions of ROS.

## The ALR mouse strain

In addition to the NOD.*Ncf1^m1J^* mouse, the alloxan-resistant (ALR) mouse provides a nice tool to explore the role of ROS in modulation of autoreactive T cell responses in T1D due to their inherent increase in antioxidant enzymes and elevated capacity to dissipate ROS.^98^,^99^ Possessing islets that are highly resistant to destruction by immune cells, ALR mice do not develop alloxan-induced diabetes; in addition, these mice are resistant to spontaneous T1D.^100^ Mapping studies performed to identify the genes responsible for their unique resistance implicated *Susp*, *Idd2*, *Idd16.2*,^101^ and the mitochondrial genome (*mtDNA*).^102^ Interestingly, *Susp*, also known as suppressor of superoxide production modulates the ability of innate immune cells, such as neutrophils and macrophages, to suppress oxidative burst upon stimulation with phorbol 12-myristate 13-acetate (PMA).^103^ In addition to modulation of superoxide production of innate immune cells within the ALR mouse strain, CD4^+^ T cells may also play an important role in T1D resistance by generating shifts in oxidative status.^99^

## Conclusion I

CD4^+^ and CD8^+^ T cells play essential roles in T1D by inducing pancreatic β-cell destruction and enhancing a proinflammatory milieu within the pancreas. CD8^+^ T cells kill via secretion of cytotoxic effector molecules, such as granzymes and perforins, as well as via activation of FasL and production of TNF-α and IFN-γ; while CD4^+^ T cells induce pathogenicity via the production of proinflammatory cytokines, which can activate macrophages or other accessory cells to destroy pancreatic β cells. Indeed, proinflammatory cytokines play essential roles in β-cell destruction and are essential for inducing efficient adaptive immune maturation. T cells display marked antigen-specific hyporesponsiveness characterized by reduced proliferation, memory, and effector functions in the absence of proinflammatory cytokines. While studies have pin-pointed IL-12 and IFN-α/β as third signal cytokines that promote efficient immunity by influencing chromatin remodeling in CD8^+^ T cells, no such link has yet been made between IL-1 and CD4^+^ T cell responses. By inducing the production of various proinflammatory cytokines via redox-dependent signaling pathways, such as MAPK, AP-1, and NF-κB, ROS are absolutely indispensable for T cell maturation. Ablation of ROS utilizing SOD mimetics that scavenge a broad range of ROS, in addition to mouse models possessing mutations in the ability to generate NOX-derived superoxide, have demonstrated the obligatory role of these highly reactive molecules in initiating a third signal for effective adaptive immune responses. Because of their potent effects on T cell activity and inhibition of T cell maturation, further studies to understand ROS and redox-dependent signaling may define a potential therapeutic target to arrest autoreactive T cell responses in T1D.

## Proinflammatory cytokines and β cells: an introduction, part II

In the previous section of this review, the third signal played by ROS and cytokines in the regulation and development of adaptive immunity was discussed. In this section, focus will be on the 25–30 years of research on the mechanisms by which cytokines cause damage to pancreatic β cells. In the early years of this field, studies focused on cytokine stimulation of free radical production and how this caused impairment in oxidative metabolism and insulin secretion by islets. More recently, studies have focused on the ability of cytokines to induce β-cell death and whether cell death occurs by necrosis or apoptosis. This section of the review will discuss how early studies on cytokine-mediated inhibition of β-cell function may influence current views regarding the mechanisms responsible for controlling the loss of β-cell viability and the type of death induced by cytokines.

## The 1980s

In seminal studies performed by Mandrup-Poulsen *et al*.^104^ in the mid-1980s, conditioned medium derived from activated peripheral blood monocytes was shown to inhibit glucose-induced insulin secretion and to cause islet damage following prolonged incubation. Interleukin-1, a macrophage-derived cytokine, was identified as the primary component of the conditioned medium that caused islet damage.^104^–^106^ These observations led to the hypothesis that cytokines, produced locally in and around islets during islet insulitis, could impair β-cell function and reduce β-cell mass during the development of autoimmune diabetes.^107^,^108^ Indeed, subsequent work showed that cytokines modify insulin secretion in a time- and concentration-dependent manner, with an initial stimulation of secretion (one to three hours) that is followed by an inhibition of secretion that begins approximately five to eight hours after cytokine treatment with maximal inhibition of secretion observed after 18 hours of cytokine treatment.^109^–^111^ Following prolonged incubations of four to seven days, cytokines induce complete islet degeneration.^104^,^112^ Yet, the inhibitory action of IL-1 is reversible; islets fully recover insulin secretory function following 15-h cytokine incubation if the cytokine is removed by washing and the islets are cultured for four days in the absence of cytokine.^109^ This reversibility is temporally limited, as a two-day incubation with IL-1 results in an irreversible inhibition of insulin secretion.^109^

The inhibition of mitochondrial oxidation is the central mechanism by which IL-1 inhibits insulin secretion. IL-1 inhibits the oxidation of glucose to CO_2_ in a temporal fashion that correlates with the inhibition of insulin secretion and is associated with a fivefold reduction in islet ATP levels.^110^,^113^,^114^ While mitochondrial oxidation is impaired, glucose utilization via glycolysis seems to be unaffected by cytokine treatment.^114^ These findings place impairment of mitochondrial metabolism and decreased cellular levels of ATP as the central mechanisms by which IL-1 damage islets. IL-1 also inhibits *de novo* protein synthesis in a temporal manner that is similar to the time-dependent inhibition of insulin secretion;^110^,^115^ yet, paradoxically, inhibitors of mRNA transcription and *de novo* protein translation protect islets and FACS-purified β cells from the damaging actions of IL-1 (Refs. 110 and 114). The above findings suggest that β cells express proteins that contribute to their own demise and that targets of their action include mitochondrial oxidation and protein translation.^116^,^117^

## The 1990s

**Figure 3:**
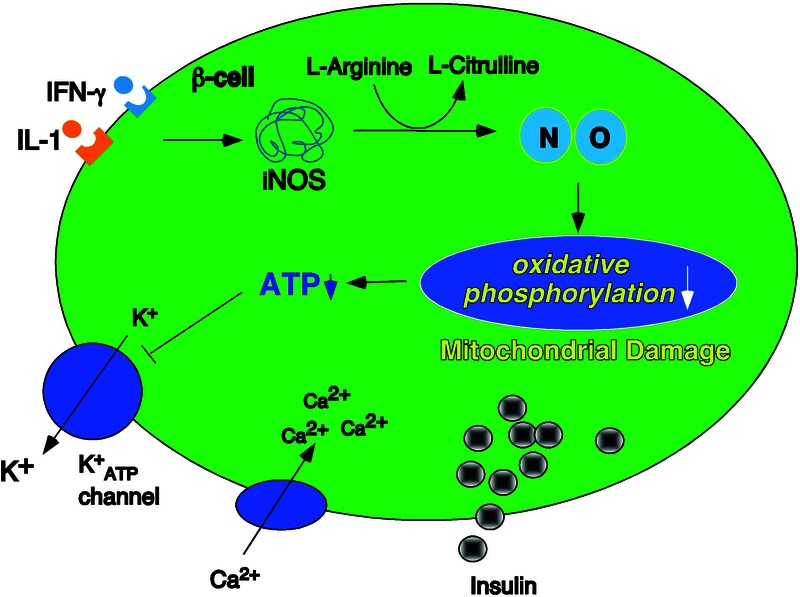
Nitric oxide mediates the inhibitory actions of cytokines on insulin secretion. Cytokines (IL-1 and IFN-γ) stimulate iNOS expression and the production of micromolar levels of nitric oxide in β cells. Nitric oxide inhibits the oxidation of glucose to CO_2_, resulting in the attenuation of glucose-dependent ATP generation. The net effect is an inhibition in the closure of ATP-sensitive potassium channels, preventing β-cell depolarization, calcium influx, and calcium-dependent insulin exocytosis.

In the late 1980s a rapidly growing community of researchers was brought together due to the emerging role of the soluble free radical gas nitric oxide in the regulation of biological phenomena. The functions ascribed to nitric oxide were diverse, from the control of vascular tone to macrophage killing of invading pathogens.^118^–^122^ In 1990, nitric oxide was made relevant to the field of β cell biology when the Green laboratory showed that IL-1 + TNF-α stimulates nitrite formation by islets and that *N*-nitro-l-arginine methyl ester, a nitric oxide synthase inhibitor, attenuates the inhibitory actions of these cytokines on insulin secretion.^123^ These observations were confirmed by two studies showing that rodent islets could make nitric oxide in response to IL-1, that nitric oxide mediates the inhibitory actions of cytokines on insulin secretion, and that the Krebs cycle enzyme aconitase was one molecular target of this free radical.^124^,^125^ In a rapid flood of new information, the expression of the three isoforms of NOS in islets was demonstrated: iNOS and nNOS in β cells, and eNOS in islet endothelial cells.^126^ Nitric oxide was shown to mediate cytokine-induced inhibition of glucose-stimulated insulin secretion by decreasing the mitochondrial oxidation of glucose to CO_2_, thereby preventing ATP accumulation.^113^,^123^,^125^,^127^ The net effect of this is an inhibition in the closure of ATP-sensitive potassium channels, preventing β-cell depolarization, calcium influx, and calcium-dependent insulin granule exocytosis.^128^,^129^ On the basis of this work, it is generally appreciated that nitric oxide is the mediator of the inhibitory actions of cytokines on insulin secretion (Fig. 3).

In addition to the functional studies on insulin secretion, the pathways regulating cytokine-stimulated iNOS expression were determined. IL-1 was shown to be sufficient for stimulating iNOS expression by rat islets and purified rat β cells,^113^,^130^ and IFN-γ was shown to prime for and potentiate the actions of IL-1.^131^–^134^ In combination, IL-1 and IFN-γ are the minimal cytokine combination required to stimulate iNOS expression in islets and β cells in most strains of mice and in human islets.^135^ Transcription factors that regulate the expression of iNOS were identified to include NF-κB in response to IL-1 and IRF-1 and STAT-1 in response to IFN-γ.^136^ Studies examining the cell–cell interactions in islets between tissue macrophages and β cells showed that the 10–15 resident macrophages found in an islet, when activated, could release sufficient amounts of IL-1 within islets to stimulate iNOS expression and nitric oxide production and to inhibit insulin secretion.^130^ These latter findings, performed in rat, mouse, and human islets, established that the concentrations of IL-1 that were used in *in vitro* studies could be generated in islets by resident macrophages, and they provided a plausible mechanism by which local cytokine production may induce β-cell injury.^107^,^137^,^138^

β cells are sensitive to short pulses in cytokine concentrations, as incubation of islets with IL-1 for one-hour results in inhibition of insulin secretion in a nitric oxide–dependent manner 18 h after cytokine removal.^110^,^139^ Further establishing nitric oxide as the primary mediator of cytokine-induced damage were the observations that the recovery of insulin secretory function could be reduced from four days to eight hours by simply inhibiting nitric oxide synthase. The addition of an iNOS inhibitor to islets (rat or human) treated for 24 h with cytokines (IL-1 or IL-1 + IFN-γ), followed by continued culture with the iNOS inhibitor and cytokine, results in a time-dependent recovery of insulin secretion, mitochondrial aconitase activity, and protein translation that is complete and maximal within eight hours.^115^,^140^ In addition to the recovery of metabolic and secretory function, islets also have the ability to repair DNA that is damaged in response to cytokine treatment.^141^,^142^ The recovery/repair from cytokine-mediated damage requires new gene expression, and nitric oxide is one stimulus that activates these repair pathways in β cells.^115^,^140^ The ability to recover from cytokine-mediated inhibition of insulin secretion is temporally limited, as there is an irreversible inhibition of insulin secretion, aconitase activity, and protein translation in islets continuously cultured for 36 h or longer with cytokines.^115^ Further, a continuous incubation for 36 h or longer commits islets to morphological degeneration.^115^,^143^ These studies establish that nitric oxide is the primary mediator of cytokine-induced islet damage. Physiologically, it is logical that the actions of cytokines on β-cell function are reversible, as islets *in vivo* are exposed to elevated levels of cytokines following infection (bacterial or viral).

## Human and rodent islet responses to cytokines

In the 2000s, the focus on insulin secretion became secondary to the effects of cytokines on islet cell viability. Along with the new focus on cell viability was the introduction of a new hypothesis that the responses of human islets to cytokines differ from the responses of rodent islets.^144^,^145^ It was suggested that human islets are more resistant to the effects of cytokines, particularly to cytokine-induced nitric oxide production, than are rodent islets.^145^–^147^ This debate, which persists today, is complicated by so-called “islet stress” and the inhibitory actions of islet stress on cytokine signaling. Islet stress, which seems to occur during islet isolation and/or islet transport, is evidenced by elevated heat shock protein (hsp) 70 expression.^148^ Islets that have been stressed (rodent or human) fail to respond to cytokines in a normal fashion;^148^–^150^ for example, IL-1 fails to activate NF-κB, and IFN-γ fails to activate Jak-STAT signaling.^150^,^151^ While the impairment of cytokine signaling is associated with enhanced hsp 70 expression, this heat shock protein is only an indicator of stress, that is, hsp 70 knockdown does not alter the inhibitory actions of stress on cytokine signaling in islets.^151^ Islets undergoing an unfolded protein response (UPR) are also similarly unaffected by cytokines.^152^ While some studies suggest that there may be differences in the response of human and rodent islets to cytokines, few studies have evaluated the level of stress or hsp 70 expression of islet preparations. The combination of IL-1 and IFN-γ is the minimal cytokine combination required to induce iNOS expression, to inhibit insulin secretion, mitochondrial aconitase activity, and protein translation, and to induce DNA damage in a nitric oxide–dependent manner.^115^,^133^–^135^,^153^ Unfortunately, in a significant proportion of human islet preparations there is enhanced hsp 70 expression, and these islets fail to respond normally to cytokines.^153^ Thus, the level of islet stress may be a major factor in complicating the interpretation of studies that have evaluated cytokine-mediated damage to human islets, with an unintended consequence being the conclusion that species differences exist in the response of islets to inflammatory cytokines.^146^

## How do cytokines kill β cells: apoptosis or necrosis?

A focus of studies over the past 10 years has been on the mechanisms by which cytokine-induced β-cell death occurs. A major outcome has been an emphasis on apoptosis as the mechanism by which cytokine treatment induces β-cell death; the majority of studies no longer examine the contributions of nitric oxide to the death process (reviewed in Refs. 154–157). To summarize many of these studies, cytokine-induced apoptosis is associated with mitochondrial directed caspase 9 activation, where cytokines stimulate enhanced expression of Bcl-2 homology 3 (BH3)-only molecules such as BIM, BAD, BID, Puma, and Noxa. Puma appears to be a primary BH3-only protein regulating apoptosis in β cells.^156^,^158^,^159^ IL-1 + IFN-γ have also been reported to stimulate the expression of the BH3-only sensitizer Harakiri or DP5.^160^ Once activated, DP5 selectively binds to and represses the activity of members of the Bcl-2 family of antiapoptotic factors, such as Mcl-1 and Bcl-Xl, leading to enhanced expression of Puma. Proapoptotic inducers, such as Puma, inhibit the interactions of the antiapoptotic Bcl-2 members with Bax and Bak, allowing these proapoptotic proteins to stimulate cytochrome c release from mitochondria, which leads to caspase 9 activation.^156^,^159^,^160^ Induction of ER stress may augment this process through the activation of the proapoptotic MAP kinase JNK,^161^,^162^ although the role of ER stress as a mediator of cytokine-induced β-cell death has been challenged.^163^,^164^ The contributions of nitric oxide to cytokine-induced β-cell apoptosis are poorly defined, as it has not been addressed in many of these studies.

In contrast to the studies reporting that cytokines induce β-cell apoptosis, other groups have reported that cytokines induce islet cell death by nitric oxide–dependent necrosis.^98^,^153^,^165^,^166^ Collier *et al.*^165^ have shown that (1) INS-832/13 β cells selected for resistance to cytokine killing are as sensitive as wild-type INS-832/13 cells to killing by proapoptotic agents; (2) enhanced expression of antiapoptotic factor AKT attenuates INS832/13 cell death in response to apoptosis-inducing agents, but not in response to cytokines; (3) knockdown of proapoptotic factor Bax attenuates INS-832/13 cell death in response to apoptosis-inducing agents but not cytokine-induced cell death; and (4) there is an absence of caspase cleavage and activity in cytokine-treated islets, while islet cell death in response to apoptosis inducers is associated with caspase activation. In addition, this group has used liquid chromatography tandem mass spectrometry (LC-MS/MS) to show that IL-1 + IFN-γ induces a metabolite profile in INS 832/13 and INS1E cells that is distinct from the profiles generated in the same cells forced to undergo apoptosis.^167^

Similar results to those of Collier *et al.*^165^,^167^ have been obtained when the effects of cytokines (IL-1 and IFN-γ) are compared to the actions known inducers of apoptosis, such as staurosporine and camptothecin.^153^ Cytokine treatment stimulates morphological changes (cell swelling) and DNA damage (TUNEL staining that covers the entire nucleus) that differs from the morphological changes induced by camptothecin (cell shrinkage, punctate nuclear TUNEL staining consistent with chromatin condensation).^153^ Cytokines failed to stimulate caspase 3 cleavage or caspase activity in INS-832/13 cells or in either rodent or human islets following 24- and 48-h incubations; however, they do stimulate the release of high mobility group box 1 protein (HMGB1) in a nitric oxide–dependent manner.^153^ HMGB1 is a marker of cellular necrosis.^168^,^169^ In contrast, camptothecin and staurosporine stimulate caspase 3 cleavage and activity in each cell type (8- to 10-fold increase) but fail to stimulate the release of HMGB1.^153^ Lastly, inhibitors of caspase 3 attenuate staurosporine- and camptothecin-induced cell death but fail to modify cytokine-stimulated β-cell death.^153^ While the work of just two groups has been highlighted,^153^,^165^,^167^ others have also suggested that cytokines can cause islet cell necrosis in a nitric oxide–dependent fashion.^136^,^166^,^170^–^173^ Additional studies provide further support for nitric oxide as the primary mediator of cytokine-induced islet cell death; for example, Eizirik *et al.*^174^ have shown that cytokine treatment results in 88% loss in viability of wild-type islets following a six-day incubation; however, cytokine treatment failed to kill islets isolated from iNOS knockouts above the level of cell death observed in control untreated islets. Also consistent with this conclusion is the development of diabetes in mice expressing iNOS under control of the insulin promoter and the prevention of disease by administration of the NOS inhibitor aminoguanidine.^175^

It is possible to reconcile the different conclusions drawn from studies examining the type of cell death induced by cytokines by looking at limitations in methodology, targets measured, and the use of positive controls. Many studies use nonspecific assays of apoptosis, such as nuclear vital dye accumulation and TUNEL staining of DNA damage.^156^,^174^ And while other studies have used more specific assays of apoptosis, such as caspase cleavage, caspase activity, and annexin staining, to assess cytokine-induced cell death, these studies have rarely been performed with known inducers of apoptosis. The use of known apoptosis inducers in control experiments has been extremely valuable in interpreting the results from some studies.^153^,^165^ For example, staurosporine stimulates a ninefold increase in caspase 3 activity in rat islets, while cytokines stimulated a 70% increase in caspase 3 activity.^153^ Under both conditions the levels of cell death (as determined using biochemical assays such as MTT or neutral red dye) are nearly identical (∼40%, Ref. 153). These findings suggest very different mechanisms of death.^153^ Confirming this conclusion, caspase inhibition attenuates staurosporine but fails to modify cytokine-induced cell death. In contrast, iNOS inhibition prevents cytokine-induced, but not staurosporine-induced β-cell death.^153^ This is also the case for assays examining caspase cleavage as an indicator of apoptosis, as high affinity antibodies allow for the detection of very low levels of caspase cleavage. For example, it is possible to detect caspase 3 cleavage by Western blot analysis in a population of cells in which less than 5% are undergoing apoptosis; however, the level of caspase cleavage is minor when compared to the level of caspase 3 cleavage induced by camptothecin (see Fig. 5 in Ref. 176).

**Figure 4:**
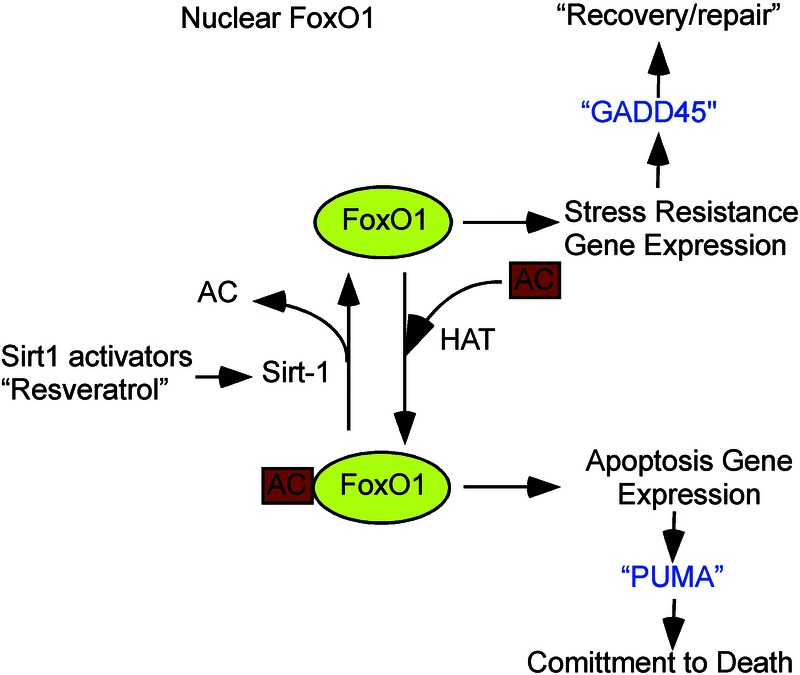
Transcriptional regulation by FOXO1 and Sirt1 in response to nitric oxide. Nitric oxide stimulates FOXO1 nuclear localization. When Sirt1 is more active, FOXO1 is deacetylated and directs the expression of protective factors such as the DNA repair gene GADD45α. When Sirt1 is less active, FOXO1 is acetylated (FOXO1-AC) and directs the expression of proapoptotic factors such as PUMA.

**Figure 5:**
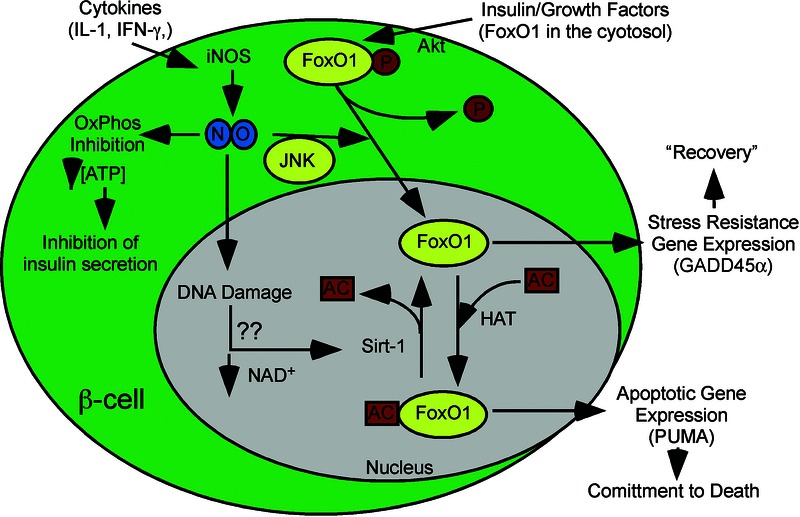
The pathways controlling the β-cell response to cytokines. Cytokines (IL-1 and IFN-γ) stimulate iNOS expression and the production of micromolar levels of nitric oxide in β cells. Nitric oxide stimulates the nuclear localization of FOXO1 that is associated with the loss of the AKT-mediated inhibitory phosphorylation. In the nucleus, FOXO1 directs a transcriptional program, as described in the legend to Figure 2. Mechanisms responsible for the regulation of Sirt1 activity are unknown, but irreversible DNA damage is associated with a commitment of β cells to death in response to cytokines. Decreased cellular levels of NAD^+^, associated with impaired mitochondrial oxidative capacity, may contribute to the regulation of this NAD^+^-dependent deacetylase. Overactivation of poly ADP-ribose polymerase (PARP) following extensive DNA damage is one potential mechanism of NAD^+^ depletion; however, we have shown that cytokines do not overactivate PARP-1 in islets.^194^

## Could cytokines kill β cells by both necrosis and apoptosis?

A key feature in cytokine-mediated β-cell death is the reversibility of this process. Generally, apoptosis is a controlled form of cell death that removes unwanted or damaged cells in the absence of inflammation. It is a process that is essentially irreversible. In contrast, necrosis is an uncontrolled form of cell death that is associated with the loss of energy homeostasis and membrane potential (permeability), with the leakage of cellular contents in an uncontrolled fashion that stimulates inflammation. If the damaging insult is removed during progression to necrotic cell death and energy balance is restored, necrosis can be prevented and the cells can recover from the injury.^177^ Beta cells have the ability to recover from the inhibitory actions of short cytokine exposures (up to 24 h) to insulin secretion, oxidative metabolism (aconitase activity), protein synthesis, and to repair the damage to DNA.^115^,^142^,^176^,^178^ However, this recovery is temporally limited, as 36-h or longer cytokine exposures result in irreversible inhibition or islet damage of each of these parameters, and the islets are committed to degeneration.^115^,^143^,^178^ Based on these findings, it may be the case that cytokines stimulate an early necrotic process where removal of the damaging insult (nitric oxide) results in a restoration of energy balance and functional recovery. However, if the injury is left unchecked and β cells no longer have the capacity to restore energy balance (i.e., following a 36-h cytokine incubation), the program shift from necrosis to apoptosis.^143^

The key regulators in this shift from necrosis to apoptosis include the rate of nitric oxide production and the extent of DNA damage. Nitric oxide is a potent and effective inhibitor of caspase activity^179^,^180^ in many cell types including β cells.^143^ Therefore, in the presence of micromolar levels of nitric oxide it would be difficult for β cells to undergo caspase-dependent apoptosis; however, when the rate of cytokine-induced nitric oxide production is decreased, which occurs following prolonged exposures of islets to cytokines (36 h or longer),^143^ the attenuation of caspase activity by nitric oxide also decreases. Following a 36-h treatment of rat islets with IL-1 or human islets with IL-1 + IFN-γ, procaspase cleavage occurs in β cells yet there is no detectable caspase activity. Addition of an iNOS inhibitor (without removing the cytokine) and continued culture for eight additional hours to inhibit residual nitric oxide production resulted in a two- to threefold increase caspase activity.^143^,^178^ Coincident with caspase 3 cleavage and unveiling of caspase activity is an irreversible damage to β-cell DNA.^143^,^178^

The mechanisms controlling the early reversible response and the later shift to apoptosis in cytokine-treated islets are beginning to be elucidated. The key regulatory factors controlling these responses include c-Jun N-terminal kinase (JNK), forkhead family of transcription factor (FOXO1), and sirtuins (Sirt1). Rapid activation of JNK by cytokines, such as IL-1, has been reported to participate in cytokine-induced β-cell apoptosis, possibly during prolonged activation of ER stress pathways in islets.^162^,^181^–^183^ Nitric oxide also activates JNK. Published studies have shown that JNK activation is required for β-cell recovery from cytokine- and nitric oxide–mediated inhibition of aconitase activity and for the repair of nitric oxide-damaged DNA in β cells.^178^,^184^ While mechanisms by which JNK participates in the recovery of mitochondrial oxidative metabolism are incompletely defined, one role that JNK plays in the repair of damaged DNA is controlling the expression of the stress sensor protein growth arrest and DNA damage (GADD) 45α.^178^ Inhibition of JNK attenuates nitric oxide–induced GADD45α expression.^178^ In addition, JNK inhibition and siRNA knockdown of GADD45α attenuate the repair of nitric oxide-damaged DNA in insulinoma cells.^178^ In many cell types, GADD45α expression is regulated by p53;^185^ however, in β cells, nitric oxide fails to activate p53 (phosphorylation or stabilization), and siRNA knockdown of p53 does not modify the ability of β cells to express GADD45α or to repair cytokine-mediated DNA damage.^178^ GADD45α expression is also regulated by FOXO1,^186^ and a study has shown that nitric oxide, supplied exogenously using chemical donors or endogenously following cytokine treatment, stimulates the nuclear localization of FOXO1 in β cells.^187^ Once in the nucleus, the posttranslational modification status determines the gene families that are regulated by FOXO1.^188^ When FOXO1 is nuclear and deacetylated, it directs a transcriptional program that includes the expression of protective genes such as GADD45α, superoxide dismutase, and PGC-1α.^189^,^190^ When FOXO1 is nuclear and acetylated (likely by CEBP or the p300 complex), the transcriptional program is proapoptotic, with enhanced expression of BH3-only genes such as Puma, BIM, and Noxa (Fig. 4, Ref. 189). Using dominant negative mutants, we have shown that FOXO1 participates in the transcriptional control of GADD45α and in the repair of β-cell DNA.^187^ Further, IL-1 stimulates the nitric oxide–dependent accumulation of Puma mRNA following 36-h of incubation or under conditions in which the damaging actions of IL-1 are irreversible.^143^ Thus, the nuclear localization and the posttranslational modifications of FOXO1 may play a primary role in determining the response of β cells to the generation of nitric oxide.

Sirt1, a NAD^+^-dependent deacetylase, plays a primary role in controlling the activity of FOXO1 in islets in response to nitric oxide. Inhibition (chemical or siRNA knockdown) of Sirt1 results in an attenuation in GADD45α mRNA and an increase in Puma mRNA in response to nitric oxide.^187^ These transcriptional changes correlate with enhanced DNA damage in response to nitric oxide and induction of caspase cleavage (to levels induced by apoptosis inducers) in response to 12-h and 24-h incubations with IL-1. In contrast, Sirt1 activation accelerates the rate of repair of nitric oxide–damaged DNA by threefold.^187^ These findings suggest that cell fate decisions in response to nitric oxide are controlled by the interactions of FOXO1 with Sirt1, and that these interactions define a transcriptional program that determines whether β cells repair cellular damage or whether the damage is too extensive and the cells therefore activate an apoptotic program.^187^

## Conclusion II

The proposed mechanisms that explain the control of β-cell responses to cytokines are shown in schematic form in Figure 5. There are many questions that have yet to be asked regarding the delicate balance between activation/inhibition of Sirt1, the cellular localization of FOXO1 and the transcriptional profiles stimulated in β cells by FOXO1 when it functions as a protective versus proapoptotic transcriptional regulator. Studies now provide a mechanism by which JNK, BH3-only proapoptotic factors such as PUMA, and caspases function in cytokine-induced β–cell apoptosis, and this mechanism includes a role for FoxO1 and Sirt1 in the regulation of these molecules.^156^,^157^,^159^,^160^ Other studies indicate that nitric oxide is a primary regulator of the β-cell response to cytokines.^98^,^165^,^174^,^191^,^192^ The role of nitric oxide is far more extensive than simply to activate the UPR and thereby augment apoptosis in response to cytokines.^193^ Future studies directed at determining how nitric oxide stimulates FOXO1 nuclear localization and the pathways controlling Sirt1 activity in β cells should shed important light on the complicated and intricate control of cytokine actions in β cells. Additional studies that examine activation of the UPR and the contributions of the UPR to protect and damage pathways in cytokine-treated islets should provide information on the physiological significance of cytokine action on β cells. Finally, further characterization of the protective pathways activated by nitric oxide could be useful to mechanistically understand how to protect β cells during replacement therapy such as transplantation.
